# Macrophage Polarization Reflects T Cell Composition of Tumor Microenvironment in Pediatric Classical Hodgkin Lymphoma and Has Impact on Survival

**DOI:** 10.1371/journal.pone.0124531

**Published:** 2015-05-15

**Authors:** Mário H. M. Barros, Priscilla Segges, Gabriela Vera-Lozada, Rocio Hassan, Gerald Niedobitek

**Affiliations:** 1 Institute for Pathology, Unfallkrankenhaus Berlin, Berlin, Germany; 2 Bone Marrow Transplantation Center, Instituto Nacional de Câncer (INCA), Rio de Janeiro, Brazil; 3 Institute for Pathology, Sana Klinikum Lichtenberg, Berlin, Germany; Boston University School of Medicine, UNITED STATES

## Abstract

Macrophages have been implicated in the pathogenesis of classical Hodgkin lymphoma (cHL) and have been suggested to have a negative impact on outcome. Most studies addressing the role of macrophages in cHL have relied on identification of macrophages by generic macrophage antigens, e.g., CD68. We have therefore conducted an *in situ* analysis of macrophage polarization in a series of 100 pediatric cHL (pcHL) cases using double staining immunohistochemistry, combining CD68 or CD163 with pSTAT1 (M1-like) or CMAF (M2-like). M1- or M2-polarised microenvironment was defined by an excess of one population over the other (>1.5). Expression of STAT1 and LYZ genes was also evaluated by RT-qPCR. Patients <14 years and EBV+ cases displayed higher numbers of CD68+pSTAT1+ cells than older children and EBV- cases, respectively (*P*=0.01 and *P*=0.02). A cytotoxic tumor microenvironment, defined by a CD8+/FOXP3+ ratio >1.5 was associated with higher numbers of CD68+pSTAT1+ (*P*=0.025) and CD163+pSTAT1+ macrophages (*P*<0.0005). Levels of *STAT1* and *LYZ* expression were associated with the numbers of CD68+pSTAT1+ macrophages. EBV+ cHL cases disclosed a predominant M1 polarized microenvironment similar to Th1 mediated inflammatory disorders, while EBV- cHL showed a predominant M2 polarized microenvironment closer to Th2 mediated inflammatory diseases. Better overall-survival (OS) was observed in cases with higher numbers of CD163+pSTAT1+ macrophages (*P*=0.02) while larger numbers of CD163+CMAF+ macrophages were associated with worse progression-free survival (PFS) (*P*=0.02). Predominant M1-like polarization as disclosed by CD163+pSTAT1+/CD163+CMAF+ ratio > 1.5 was associated with better OS (*P*= 0.037). In conclusion, macrophage polarization in pcHL correlates with prevalent local T cell response and may be influenced by the EBV-status of neoplastic cells. Besides, M1-like and M2-like macrophages displayed differential effects on outcome in pcHL.

## Introduction

Hodgkin and Reed-Sternberg (HRS) cells are typical for classical Hodgkin lymphoma (cHL) in both, children and adults [1]. However, microenvironment composition of cHL differs between pediatric and adult cases [2–4]. In adult cHL Epstein-Barr virus (EBV) positive-cases may be associated with a dysfunctional senescent immune system and viral reactivation, at least in old patients [5]. By contrast, in children, EBV-associated cases are associated with primary infection, and the tumor microenvironment is characterized by a predominantly cytotoxic/Th1 profile [3].

It has been reported that high numbers of tumor-associated macrophages (TAM) are associated with poor outcome in adult cHL [6–9]. We have previously shown that in pediatric cHL not all CD68+ macrophages are also CD163+, and that the prognostic role of these cells is influenced by the EBV-status [4]. These observations have led us to hypothesize that in the tumor microenvironment of pediatric cHL, macrophages may represent a heterogeneous cell population.

In a simplified view of macrophage activation, macrophages may exhibit two functional states, which represent the extremes of a continuum of activation states, one with a pro-inflammatory phenotype, the ability to promote T helper 1 (Th1) immune responses and tumoricidal activity (M1 macrophages) and another with regulatory functions in tissue repair, remodelling and promotion of Th2 immune response (M2 macrophages) [10–12]. One important function of a subset of M2 macrophages is the production IL10 [13–18]. Two transcription factors are differentially expressed by polarized macrophages; STAT1 is expressed in M1 macrophages, it is upregulated in response to types I, II or III interferon and its phosphorylated form (pSTAT1) binds to the promoter region of interferon-stimulated genes [12,19–21]. CMAF, an essential transcription factor for interleukin (IL) -10 production, is expressed in M2 macrophages committed to IL-10 production [22–24]. CD163 has been considered a M2-specific marker in macrophages [25–27]. Recently, we have demonstrated that the combined use of a macrophage marker, such as CD68 or CD163, together with pSTAT1 or CMAF can be used to identify M1- or M2-polarized macrophages, respectively. The results of our analysis of various immunologically mediated diseases suggested that CD163 should not be regarded as a specific M2-marker [28,29].

Here, we have examined macrophage populations in the tumor microenvironment of pediatric cHL using double-labeling immunohistochemistry and gene expression analysis. Additionally, we have studied the impact of these macrophages on the outcome of pediatric cHL.

## Material and Methods

### Patients

One hundred HIV-negative children and adolescents (up to 18 year old) diagnosed with cHL at the Instituto Nacional do Câncer (INCA, Brazil) between 1999 and 2006 were included in this study. The study was approved by the INCA Ethics Committee and has been conducted according to the principles expressed in the Declaration of Helsinki. All ethical recommendations proposed by INCA Ethics Committee were adopted and written informed consent was obtained.

The histological and clinical features of these cases have been described previously [3]. Latent EBV infection has been investigated previously in all cHL cases [2,30]. Patients were classified into two age groups (≤ 14 years vs. >14 years) [31–35]. All patients were treated according to standard pediatric protocols [3].

In order to compare cHL macrophage polarization with non-neoplastic inflammatory diseases, we additionally compared our data with previously published results of an analysis of 17 tonsils with a diagnosis of acute infectious mononucleosis (IM) and 11 cases of Crohn´s disease (CD) representing diseases with a predominant cytotoxic/Th1 immune response [36,37]. As predominant Th2 immune response disease, 11 cecal appendices with oxyuriasis [38], 10 allergic nasal polyps with prominent eosinophilia [10,39], 10 skin biopsy samples showing wound healing [40,41] and 9 skin samples with foreign body granulomas were included [42]. All cases were selected from the archives of the Institute of Pathology, Unfallkrankenhaus Berlin, as previously described [28].

### Immunohistochemistry

Construction of tissue microarray (TMA) blocks including all cHL cases has been described previously [3]. For each patient, two 1-mm-diameter cores, selected from two different representative tumor areas rich in Hodgkin and Reed-Sternberg cells, were included. All cases showed cores with representative tumor microenvironment and high numbers of HRS cells (from 10 to 178 neoplastic cells/mm^2^, median: 70 cells/mm^2^). Buffers used for antigen retrieval and primary antibodies are listed in (Table A in S1 File). The double immunohistochemistry methodology has been described previously [28]. pSTAT1 (polyclonal, Santa Cruz Biotechnology, Dallas, USA) or CMAF (M-153, Santa Cruz Biotechnology, Dallas, USA) antibodies were used as first primary antibodies and the detection of bound antibodies was performed using ZytoChem Plus HRP polymer kit (Zytomed Systems, Berlin, Germany), employing diaminobenzidine (DAB) as chromogen. CD68 (clone PGM1, Dako, Gloustrup, Denmark) or CD163 (clone 10D6, Novocastra, Wetzlar, Germany) antibodies were incubated posteriorly, followed by detection with AP Polymer System (Zytomed Systems, Berlin, Germany), employing Blue Alkaline Phosphatase substrate kit (Vector Laboratories, Burlingame, USA) as substrate. The sections were not counterstained.

### Thresholds

Computer assisted microscopical analysis was performed as described previously [2,3]. For each cell subset, the 50^th^ and 75^th^ percentiles were used to categorize the intensity of cellular infiltration.

As yet, no thresholds have been defined for considering immune responses as M1-polarised or M2-polarised. We have therefore arbitrarily defined those cases with a M1/M2 ratio of >1.5 as M1 polarized and those with a M2/M1 ratio >1.5 as M2-polarized. Cases with lower ratios were considered as non-polarized. These calculations were done separately for CD68-positive and CD163-positive cells. This approach has proved useful and valid in our previous study of inflammatory diseases [28]. This strategy is especially important for the comparison of tissue microenvironment of cHL (which is rich in immune cells) and the microenvironment of inflammatory diseases included in this study, which may contain other cells, e.g., epithelial cells, to avoid bias that would be introduced by comparing absolute macrophage numbers of per mm^2^.

Cases displaying no labeled cell in both cores were considered not evaluable for technical reasons.

### Gene Expression Analysis

Gene expression levels of lysozyme (*LYZ*) and signal transducer and activator of transcription 1 (*STAT1*) genes, which have been previously associated to a macrophage signature in cHL [43,44], were evaluated by reverse transcriptase quantitative real-time PCR (RT-qPCR) assays. Eighty-four cases had good-quality RNA to perform quantitative analysis, i.e. Cq <35 cycles in reference gene amplifications, and amplification plots compatible with a sigmoid curve.

RNA was extracted from FFPE lymph node sections with the Master Pure RNA purification Kit (Epicentre, Madison, USA) as described previously [45]. First-strand cDNA was synthesized from 0.5 μg RNA using the High Capacity cDNA Archive kit, followed by a pre-amplification step using TaqMan PreAmp Master Mix (Applied Biosystems, Massachusetts, USA). RT-qPCR assays were performed using an ABI Prism 7000 Sequence Detection System (Applied Biosystems, Massachusetts, USA) with specific TaqMan Gene Expression Assays (Applied Biosystems, assays ID Hs00234829_m1 for *STAT1* and Hs00426231_m1 for *LYZ*) [45]. Quantification was performed using the 2^ΔCq algorithm, with glucuronidase beta (*GUSB*) and hydroxymethylbilane synthase (*HMBS*) as reference genes. Each measurement was performed in duplicate and quantified by Cq-value with fixed thresholds; replicates with standard deviation (SD) up to 0.15 cycles were accepted. In each run, Cq >35 cycles for reference genes lead to repeat analysis and ultimately to exclusion from analysis. Analyses were performed with the GenEx software (MultiD Analyses AB, Göteborg, Sweden).

### Statistical Analysis

To verify association between variables, Pearson’s chi-square, Fisher’s exact test, Mann-Whitney test and Spearman´s rank correlation were used as described previously [2–4,28,30]. Progression-free survival (PFS) was the interval (in months) from diagnosis to progression at any time, relapse from complete response, or initiation of new, previously unplanned treatment or to the last follow-up in the patients with treatment success. Overall survival (OS) refers to the interval (in months) from the diagnosis to death or last follow-up. Survival distributions were estimated by the Kaplan-Meier method and differences were compared using log-rank test. Data were analyzed using SPSS 13.0.

## Results

Clinical and histological features of the cHL cases have been described previously [2,3] and are summarized in (Table B in S1 File). In brief, age at diagnosis ranged from 3 to 18 years (median 14 years). Nodular sclerosis (NS) was the predominant subtype (69/100, 69%), followed by mixed cellularity (MC) (23/100, 23%) [2,3]. EBV was detected in HRS cells from 43 cases and no association with age group was observed [2,3]. Distribution of lymphocytes and macrophages in the tumor microenvironment in relation to age group, histology, EBV-status and their prognostic impact have been reported previously [2–4] and are summarized in Table C in S1 File.

### Characterization of Macrophage Subsets

As described previously [28], we used co-expression of pSTAT1 together with CD68 or CD163 to identify M1-polarized macrophages, while co-expression of CMAF in conjunction with CD68 or CD163, was used to identify M2-polarized macrophages.

**Fig 1:**
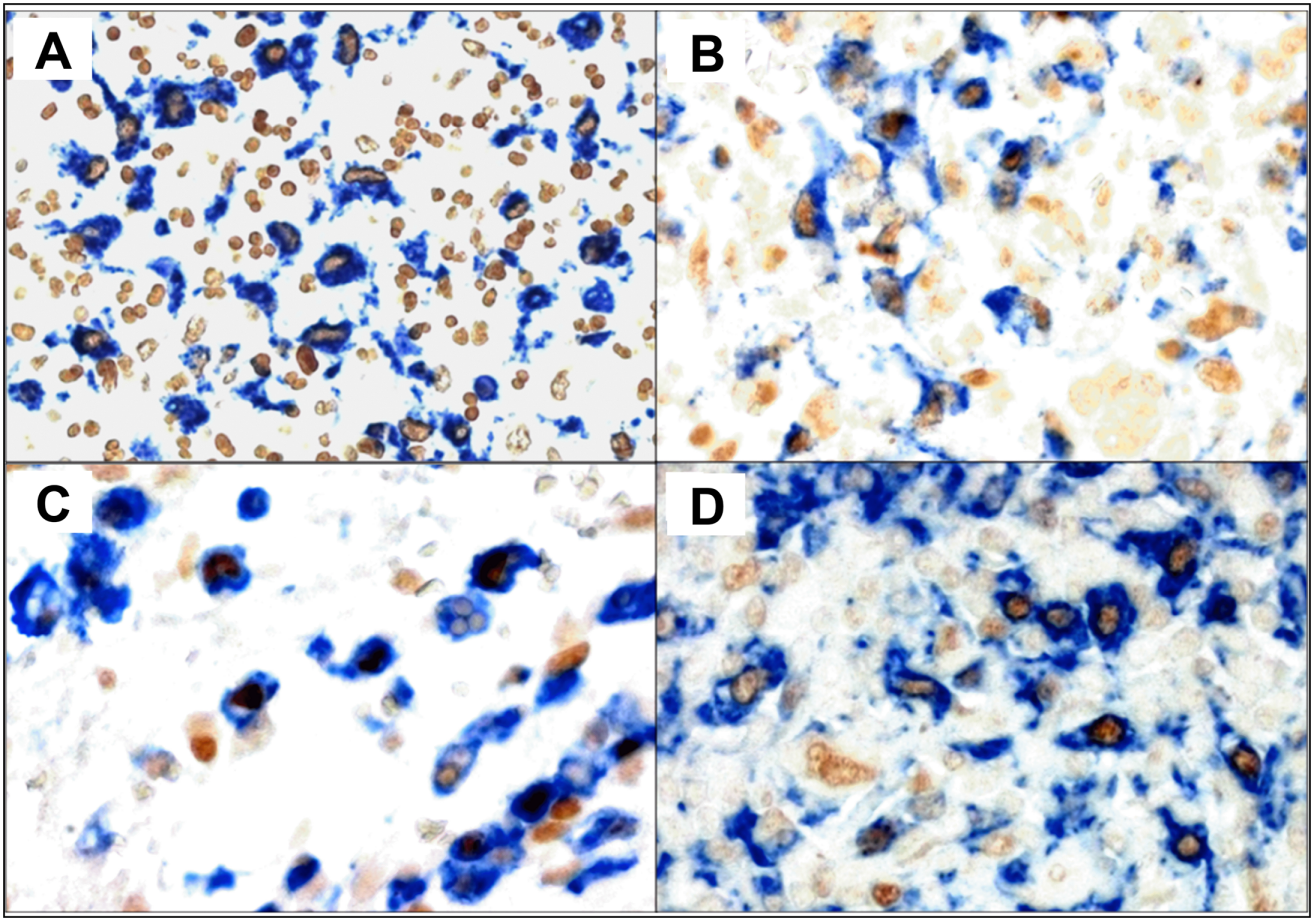
Examples of immunostains used to identify M1-like and M2-like macrophages in classical Hodgkin lymphoma. Expression of CD68 or CD163 is indicated by blue cytoplasmic/membranous staining. The expression of transcription factors pSTAT1 and CMAF is indicated by brown nuclear staining. Examples of cases with high numbers of M2-like macrophages are shown in A, (CD68+CMAF+ macrophages) and in C, (CD163+CMAF+ macrophages). Examples of cases with large numbers of M1-like macrophages are shown in B, (CD68+pSTAT1+ macrophages) and in D (CD163+pSTAT1+ macrophages) (original magnification: 400x). The sections were not counterstained.

In our cHL cases, a variable composition of intratumoral macrophage sub-populations was observed, without distinct distribution patterns of labeled cells in the microenvironment (Fig 1). Cells co-expressing either macrophage markers together with pSTAT1 or CMAF and cells expressing macrophage markers without simultaneous expression of the transcription factor under analysis were observed in all cases. In addition, cells expressing only pSTAT1 or CMAF but no macrophage antigens were also observed probably partly representing T cell subsets (S2 Fig). The results of quantitative analyses of CD68+pSTAT1+, CD68+CMAF+, CD163+pSTAT1+ and CD163+CMAF cells are summarized in Table 1.

**Table 1:** Description of macrophage subpopulations in the tumor microenvironment of pediatric classical Hodgkin lymphoma and their association with progression free survival and overall survival.

Variable	Labeled Cells /mm^2^	Cases Analyzed (%)	5-year PFS Rate (%)	Univariate Analysis (*P*) for PFS	5-year OS Rate (%)	Univariate Analysis (*P*) for OS
**CD68+pSTAT1+ (cells/mm** ^**2**^ **)**						
Range	0 to 214					
(Mean / Median)	(66.89 / 49)					
≤ 49 (50^th^ percentile)		36/71 (50.7)	72.7	0.9	85.3	0.9
> 49 (50^th^ percentile)		35/71 (49.3)	71.9		85.3	
≤ 99 (75^th^ percentile)		54/71 (76.1)	70	0.5	84.6	0.9
> 99 (75^th^ percentile)		17/71 (23.9)	80		87.5	
**CD68+CMAF+(cells/mm** ^**2**^ **)**						
Range	0 to 143					
(Mean / Median)	(45.86 / 35)					
≤ 35 (50^th^ percentile)		37/71 (52.1)	77.1	0.3	88.6	0.3
> 35 (50^th^ percentile)		34/71 (47.9)	66.7		81.8	
≤ 76 (75^th^ percentile)		54/71 (76.1)	76	0.2	90	0.038
> 76 (75^th^ percentile)		17/71 (23.9)	60		69	
**CD163+pSTAT1+ (cells/mm** ^**2**^ **)**						
Range	0 to 155					
(Mean / Median)	(34.18 / 17)					
≤ 17 (50^th^ percentile)		31/67 (46.3)	65.5	0.1	80	0.02
> 17 (50^th^ percentile)		36/67 (53.7)	84.4		100	
≤ 52 (75^th^ percentile)		51/67 (76.1)	71.4	0.1	88.8	0.3
> 52(75^th^ percentile)		16/67 (23.9)	91.7		100	
**CD163+CMAF+ (cells/mm** ^**2**^ **)**						
Range	0 to 184					
(Mean / Median)	(44.56 / 29)					
≤ 29 (50^th^ percentile)		43/81 (53.1)	86.8	0.02	92.5	0.09
> 29 (50^th^ percentile)		38/81 (46.9)	63.9		78.4	
≤ 76 (75^th^ percentile)		18/81 (22.2)	80.4	0.05	91.5	0.009
> 76 (75^th^ percentile)		63/81 (77.8)	61.1		66.7	

PFS: progression free survival. OS: overall survival.

Comparing the absolute numbers of each cell population, we observed higher numbers of cells co-expressing CD68 and pSTAT1 or CMAF (median 48 and 35 cells/mm^2^, respectively) than cells co-expressing CD163 and pSTAT1 or CMAF (median 23 and 29 cells/mm^2^, respectively) (Table 1). This indicates that not all CD68+ cells are also CD163+, and that CD163 is possibly expressed by a subset or subsets of macrophages independently of the question of polarization.

**Fig 2:**
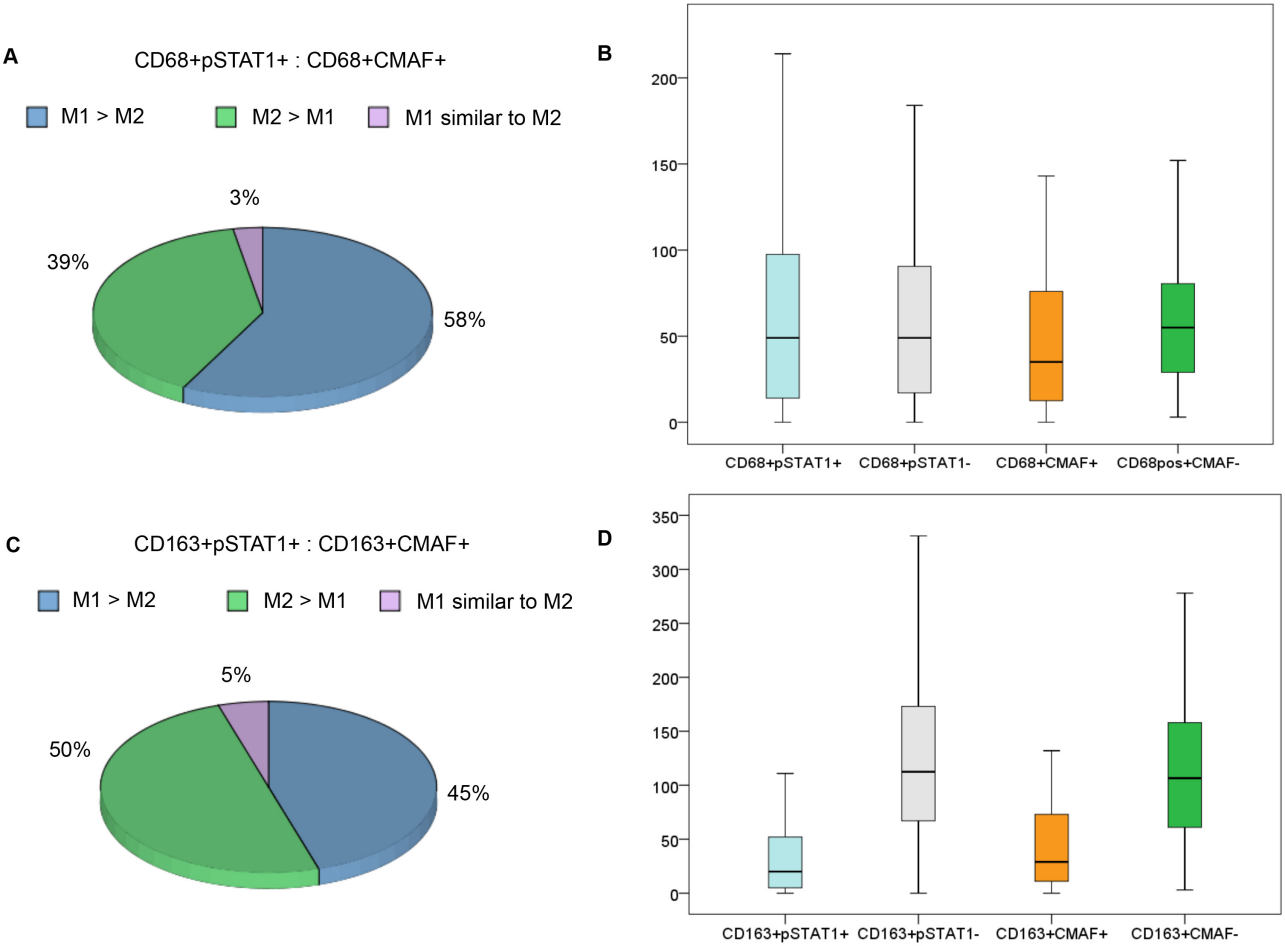
Pie charts showing the kind of macrophage polarization in pediatric classical Hodgkin lymphoma, considering CD68 (A) or CD163 (C) as macrophage markers. Box-plot graphs show the numerical distribution of CD68+pSTAT1+, CD68+pSTAT1-, CD68+CMAF+ and CD68+CMAF- cells/mm2 (B), as well as CD163+pSTAT1+, CD163+pSTAT1-, CD163+CMAF+ and CD163+CMAF- cells/mm2 (D).

**Fig 3:**
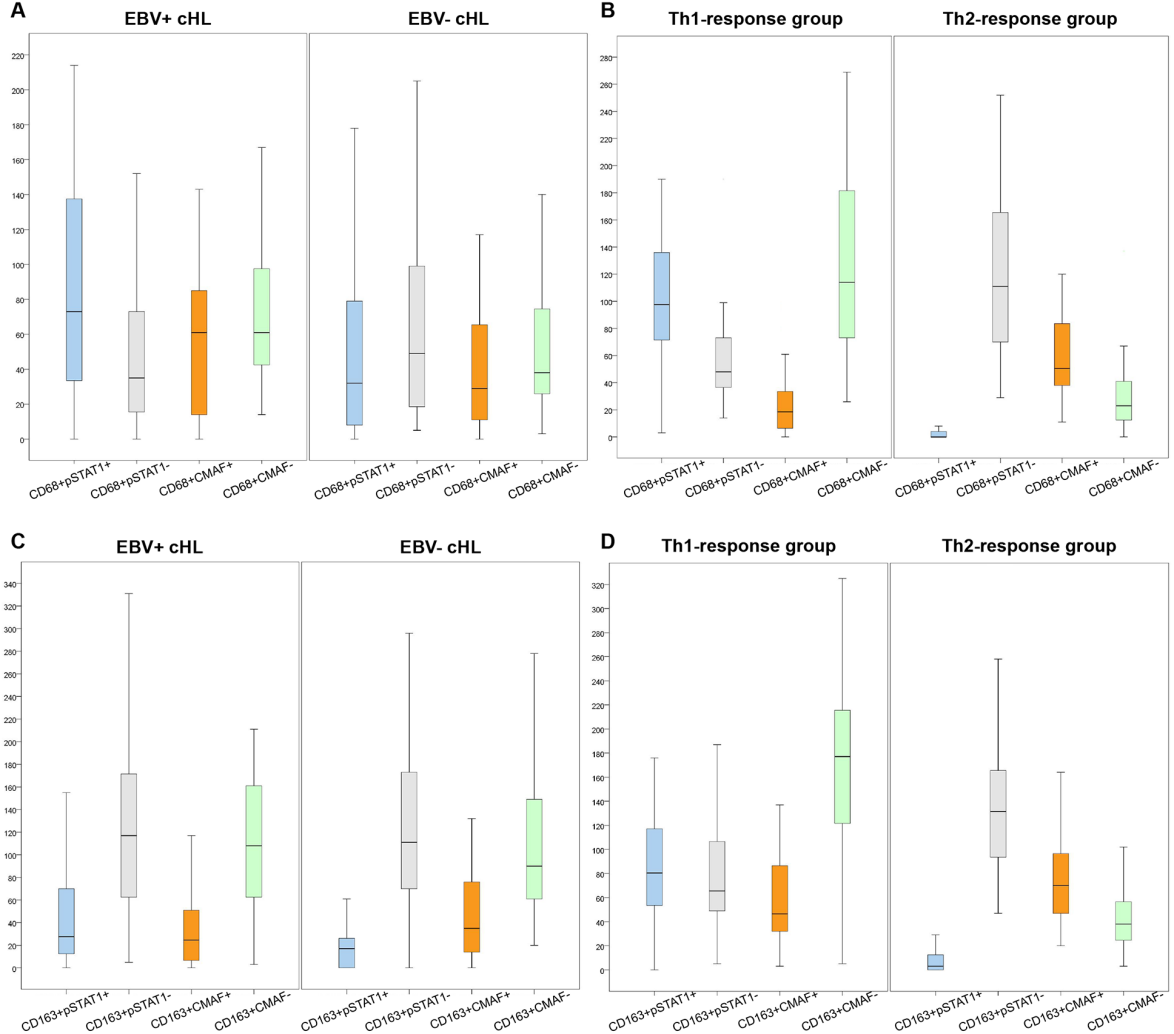
Box-plot graphs show the numerical distribution of CD68+pSTAT1+, CD68+pSTAT1-, CD68+CMAF+ and CD68+CMAF- cells/mm2 according to EBV-status (A) and Th-response group (B). The numerical distribution of CD163+pSTAT1+, CD163+pSTAT1-, CD163+CMAF+ and CD163+CMAF- cells/mm2 according to EBV-status and Th-response group is shown in C and D, respectively.

Using CD68 as macrophage marker, 58% of cases (41/71) displayed M1-like polarization (CD68+pSTAT1+ / CD68+CMAF+ >1.5), 39% (28/71) M2-like polarization (CD68+pSTAT1+ / CD68+CMAF+ <0.75) and 3% (2/71) of cases showed similar numbers of M1- and M2-like macrophages (Figs 2 and 3). Considering CD163+ cells, 45% of cases (28/62) displayed M1-like polarization (CD163+pSTAT1+ / CD163+CMAF+ >1.5), 50% (31/62) M2-like polarization (CD163+pSTAT1+ / CD163+CMAF+ <0.75) and 5% (3/62) of cases showed similar numbers of M1- and M2-like macrophages (Figs 2 and 3).

### Macrophage polarization is related to local T cell polarization

A cross-talk between innate and adaptive immune system is known to occur in immune responses (19,20) and bidirectional interactions between macrophages and lymphocytes have been described in cancer [10,46]. Since a direct, albeit complex, relationship between Th immune response polarization and macrophage polarization exists [46], we decided to investigate if a Th1/cytotoxic predominant tumor microenvironment might be associated with higher numbers of M1-like macrophages. For this purpose, we have arbitrarily defined a ratio of CD8+ cells over FoxP3+ cells of >1.5 as indicating a predominantly cytotoxic microenvironment using previously published results obtained in this case series [3].

Using this approach, a cytotoxic tumor microenvironment was associated with higher absolute numbers of CD68+pSTAT1+ (median 73 cells/mm^2^ for CD8+/FOXP3+ ratio >1.5 vs. median 12 cells/mm^2^ for CD8+/FOXP3+ ratio <0.75; P = 0.025, Mann-Whitney) and CD163+pSTAT1+ macrophages (median 26 cells/mm^2^ for CD8+/FOXP3+ ratio >1.5 vs. median 1 cell/mm^2^ for CD8+/FOXP3 ratio <0.75; P< 0.0005, Mann-Whitney).

Additionally, cases with CD68+pSTAT1+ / CD68+CMAF+ ratio >1.5 disclosed higher absolute numbers of TIA1+ lymphocytes (median 143 cells/mm^2^ vs. 55 for M2>M1; *P* = 0.001). Furthermore, cases with more CD163+pSTAT1+ than CD163+CMAF+ macrophages (ratio >1.5) showed higher absolute numbers of CD3+ lymphocytes (median 785 cells/mm^2^ vs. 604 M2>M1; *P* = 0.01) and CD8+ lymphocytes (median 264 cells/mm^2^ vs. 132 for M2>M1; *P* = 0.002). Detailed results are provided in Table 2.

**Table 2:** Numbers of lymphocytes and monocytes according to M1/M2-like macrophage ratio.

MACROPHAGE POLARIZATION^a^	CELL POPULATIONS^a^								
	CD3+ (median)	CD4+ (median)	FOXP3+ (median)	TBET+ (median)	CD8+ (median)	TIA1+ (median)	GRZB+ (median)	CD20+ (median)	CD14+ (median)
**M1 > M2**									
CD68+pSTAT1+ >	225 to 1170	11 to 551	1 to 492	3 to 202	11 to 847	10 to 396	1 to 451	3 to 885	2 to 524
CD68+CMAF+	(748)	(187)	(56)	(38)	(203)	(143)	(17)	(238)	(88)
**M2 > M1**									
CD68+ CMAF+ >	70 to 1187	14 to 624	1 to 457	5 to 167	11 to 434	2 to 319	1 to 99	5 to 879	1 to 152
CD68+pSTAT1+	(595)	(143)	(82)	(26)	(135)	(55)	(14)	(196)	(36)
*P* ^b^	0.07	0.3	0.9	0.2	0.06	0.001	0.3	0.2	0.004
**M1 > M2**									
CD163+pSTAT1+ >	316 to 1187	29 to 551	1 to 351	2 to 264	23 to 847	10 to 396	2 to 451	20 to 879	4 to 524
CD163+CMAF+	(801)	(206)	(68)	(32)	(281)	(103)	(14)	(208)	(83)
**M2 > M1**									
CD163+CMAF+ >	70 to 1170	3 to 624	1 to 513	5 to 167	11 to 527	7 to 396	1 to 211	5 to 885	1 to 275
CD163+PSTAT1+	(630)	(108)	(100)	(32)	(132)	(59)	(20)	(190)	(44)
*P* ^b^	0.01	0.05	0.1	0.4	0.002	0.4	0.9	0.7	0.2

^a)^ The numbers represent labeled cells /mm^2^.

^b)^ *P* values are from Mann-Whitney test.

To validate these results using another methodology, we decided to evaluate if expression levels of *STAT1* and *LYZ*, two genes related to macrophage-signatures [43,44] and possibly to M1 polarization [47], might be associated with the tumor microenvironment profile.

A positive correlation was observed between expression of *STAT1* and of *LYZ* (rho 0.73, *P*< 0.001, Spearman’s correlation) confirming the consistency of the gene expression signature. Next, we checked if *STAT1* and *LYZ* expression levels were correlated with the numbers of M1-like macrophages. *STAT1* levels increased with the numbers of CD68+pSTAT1+ and CD68+CMAF- cells (rho 0.479 and 0.40, respectively; *P*<0.001 for both, Spearman’s correlation) (S2 Fig). *LYZ* expression level also exhibited a positive correlation with the numbers of CD68+pSTAT1+ (rho 0.346; *P* = 0.0043, Spearman´s correlation) and CD68+CMAF- macrophages (rho 0.317; *P* = 0.009, Spearman´s correlation) (S2 Fig). In line with this, *STAT1* and *LYZ* expression levels were directly correlated with the number of cytotoxic T cells (TIA1+ and Granzyme B+ cells; *P*< 0.001 and *P* = 0.013 for *STAT1*, respectively; both *P*< 0.01 for *LYZ*. Spearman´s correlation).

From these results, we conclude that macrophage polarization and the absolute numbers of M1-like macrophage are related to and possibly influenced by the T cell composition of the tumor microenvironment.

### EBV+ cHL displays M1-like polarization reminiscent of Th1-response inflammatory disorders

As EBV appears to play an important role in the modulation of lymphocyte composition of the tumor microenvironment in pediatric [3,48], and adult cHL [49] and given the above results that indicate that lymphocyte compositions may be associated with macrophage polarization, we decided to evaluate the influence of HRS cell EBV status on macrophage-polarization in cHL.

At the gene expression level, we observed that EBV+ cases showed higher expression levels of *STAT1* (2.72±0.92 vs. 2.01±1.12 to EBV- cases; *P* = 0.023, Mann-Whitney test) and *LYZ* (3.62±1.39 vs. 2.73±1.6 to EBV- cases; *P* = 0.011 for *LYZ*, Mann-Whitney test) (S3 Fig).

At the cellular level, we observed that higher absolute numbers of CD68+pSTAT1+ macrophages were found in EBV+ cases (median 73 cells/mm^2^ vs. median 32 cells/mm^2^ in EBV- cases; *P* = 0.02, Mann-Whitney) (S3 Fig). This was also observed for CD163+pSTAT1+ macrophages (median 29 cells/mm^2^ in EBV+ vs. median 17 cells/mm^2^ in EBV- cases; *P* = 0.06, Mann-Whitney) (Fig 3 and S3 Fig).

When the macrophage ratios were considered, 64.5% (20/31) of EBV+ cHL cases showed CD68+pSTAT1+ / CD68+CMAF+ ratio > 1.5 (P = 0.2, likelihood ratio). Considering the CD163+pSTAT1+ / CD163+CMAF+ ratio > 1.5, 50%(14/28) of these EBV+ cases showed M1-like polarization (P = 0.04, likelihood ratio) (Fig 3 and S3 Fig).

Additionally, we decided to test the hypothesis that macrophage composition in pediatric EBV+ cHL would be similar to macrophage composition in prototypical conditions with predominant cytotoxic/Th1 immune response studied previously [28]. For this, we compared the macrophage composition in EBV- cHL and EBV+ cHL with conditions showing predominant cytotoxic/Th1 or Th2 immune responses taking into consideration the ratio of M1 and M2 macrophages per case.

A detailed analysis of macrophage polarization in these benign conditions has been published previously [28], which confirmed the presence of high numbers of CD68+pSTAT1+ and CD163+pSTAT1+ macrophages in Th1-response group diseases, compared with higher numbers of CD68+CMAF+ and CD163+CMAF+ in Th2-response group diseases (Table F in S1 File).

Comparison of cHL grouped according to EBV status with these non-neoplastic diseases revealed that more CD68+pSTAT1+ than CD68+CMAF+ macrophages (ratio >1.5) were observed in 89% (25/28) of Th1-response group cases and in 64.5% (20/31) of EBV+ cHL cases, but not in any of the 40 Th2-response group cases (P< 0.0005, X^2^) (Fig 3 and Table D in S1 File). Similarly, more CD163+pSTAT1+ than CD163+CMAF+ macrophages (ratio >1.5) were observed in 64.3% (18/28) of Th1-response group cases and in 50% (14/28) of EBV+ cHL, but not in any of the 40 Th2-response group cases (P< 0.0005, X^2^) (Fig 3 and S3 Fig).

Considering the EBV- group, more CD68+CMAF+ than CD68+pSTAT1+ macrophages (ratio >1.5) were observed in 100% (40/40) of Th2-response group, in 41% of EBV- cHL cases and in only 3.6% (1/28) of Th1-response group cases (P< 0.0005, X^2^). (Fig 3 and Table E in S1 File). Furthermore, more CD163+CMAF+ than CD163+pSTAT1+ macrophages (ratio >1.5) were observed in 100% (40/40) of Th2-response group cases, in 60.6% (20/33) of EBV- cHL cases and in only 10.7% (3/28) of Th1-response group cases (P< 0.0005, X^2^) (Fig 3 and Table E in S1 File).

These results highlight two aspects of the neoplastic microenvironment. The comparison of cHL with non-neoplastic conditions representative of Th1- and Th2-predominant immune responses suggests that the extent of macrophage polarization in cHL may be weaker than that observed in inflammatory conditions, in keeping with the immune dysfunction underlying cHL pathogenesis [50]. Analyzed in this context, pediatric EBV+ cHL cases displayed macrophage composition more similar to Th1-response group cases while macrophage composition in EBV- cHL was closer to Th2-response group cases.

### Macrophage polarization is associated with clinical and histological characteristics in cHL

Higher absolute numbers of CD68+pSTAT1+ macrophages were associated with young age (median 73 cells/mm^2^ in <14y vs. median 30 cells/mm^2^ in >14y; *P* = 0.01, Mann-Whitney test) and NS grade (G) I (median 26 cells/mm^2^ vs. median 14 cell/mm^2^ in NS GII; *P*< 0.005, Mann-Whitney test) (S3 Fig).

Regarding the numbers of CD163+pSTAT1+ macrophages, higher absolute numbers of these cells were associated with male gender (median 26 cells/mm^2^ vs. median 14 cells/mm^2^ in females; *P* = 0.009, Mann-Whitney test) (S3 Fig).

Considering macrophage polarization, M1-like polarization as disclosed by CD68+pSTAT1+ / CD68+CMAF+ ratio >1.5 was associated with MC subtype (83.3% of MC cases showed M1 > M2; *P* = 0.024, Fisher´s exact test) and NS GI (69% of NS GI cases showed M1>M2, compared with only 37.5% of NS GII cases; *P* = 0.028, X^2^). No other associations were observed (Table 3).

**Table 3:** Clinical and histological variables according to M1/M2-like macrophage ratio.

	MACROPHAGES			MACROPHAGES		
VARIABLE	CD68+pSTAT1+ > CD68+CMAF+^a^	CD68+CMAF+ > CD68+pSTAT1+^a^	*P*	CD163+pSTAT1+ > CD163+CMAF+^b^	CD163+CMAF+ > CD163+pSTAT1+^b^	*P*
	(M1 > M2)	(M2 > M1)		(M1 > M2)	(M2 > M1)	
**Age, years**						
≤ 14	16 (53.3)	14 (46.7)		11 (40.7)	16 (59.3)	
> 14	25 (65)	14 (35.9)	0.3	17 (53.1)	15 (46.9)	0.3
**Gender**						
Male	31 (66)	16 (34)		25 (59.5)	17 (40.5)	
Female	10 (45.5)	12 (54.5)	0.1	3 (17.6)	14 (82.4)	0.004
**Stage**						
I and II	24 (55.8)	19 (44.2)		19 (51.4)	18 (48.6)	
III and IV	13 (59)	9 (41)	0.8	7 (39)	11 (61)	0.3
**Extranodal disease**						
Yes	4 (50)	4 (50)		2 (33.3)	4 (66.7)	
No	33 (58)	24 (42)	0.7	24 (49)	25 (51)	0.6
**B symptoms**						
Yes	24 (63.2)	14 (36.8)		15 (47)	17 (53)	
No	13 (48)	14 (52)	0.2	11 (47.8)	12 (52.2)	0.9
**Clinical Presentation**						
Favorable	20 (62.5)	12 (37.5)		17 (60.7)	11 (39.3)	
Unfavorable	17 (51.5)	16 (48.5)	0.3	9 (33.3)	18 (66.7)	0.04
**Histopathological Diagnosis**						
Mixed cellularity	15 (83.3)	3 (16.7)		6 (46.2)	7 (53.8)	
Not- Mixed cellularity	26 (51)	25 (49)	0.024	22 (47.8)	24 (52.2)	1

^a)^ In 2 cases, the numbers of CD68+pSTAT1+ and CD68+CMAF+ macrophages were similar.

^b)^ In 3 cases, the numbers of CD163+pSTAT1+ and CD163+CMAF+ macrophages were similar.

M1-like polarization as defined by CD163+pSTAT1+ / CD163+CMAF+ ratio >1.5 was associated with male gender (60% of males showed M1>M2, compared with 17% of females; *P* = 0.004, Fisher´s exact test), favorable clinical presentation (60.7% vs. 33.3% in unfavorable clinical presentation; *P* = 0.04, X^2^) and absence of mediastinal mass (73% of patients without mediastinal mass showed M1>M2 and 67% of patients with mediastinal mass had M2>M1; *P* = 0.005, Fisher´s exact test). No other associations were observed (Table 3).

### CD163+CMAF+ but not CD163+pSTAT1+ Macrophages are Associated with Worse Outcome

Our previous results showed that high numbers of CD163+ macrophages were associated with worse PFS mainly in pediatric patients with EBV- cHL, while in EBV+ cHL, high numbers of CD163+ macrophages were not associated with a worse outcome [4]. We hypothesized that M1-like and M2-like macrophages may have different roles in tumor biology and therapy response. As currently there is no cHL animal model and *in vitro* studies with cHL cell lines do not represent the *in vivo* complexity of cHL microenvironment, survival analyses may be used to examine possible contributions of M1- and M2-like macrophages to the immune response against HRS cells.

**Fig 4:**
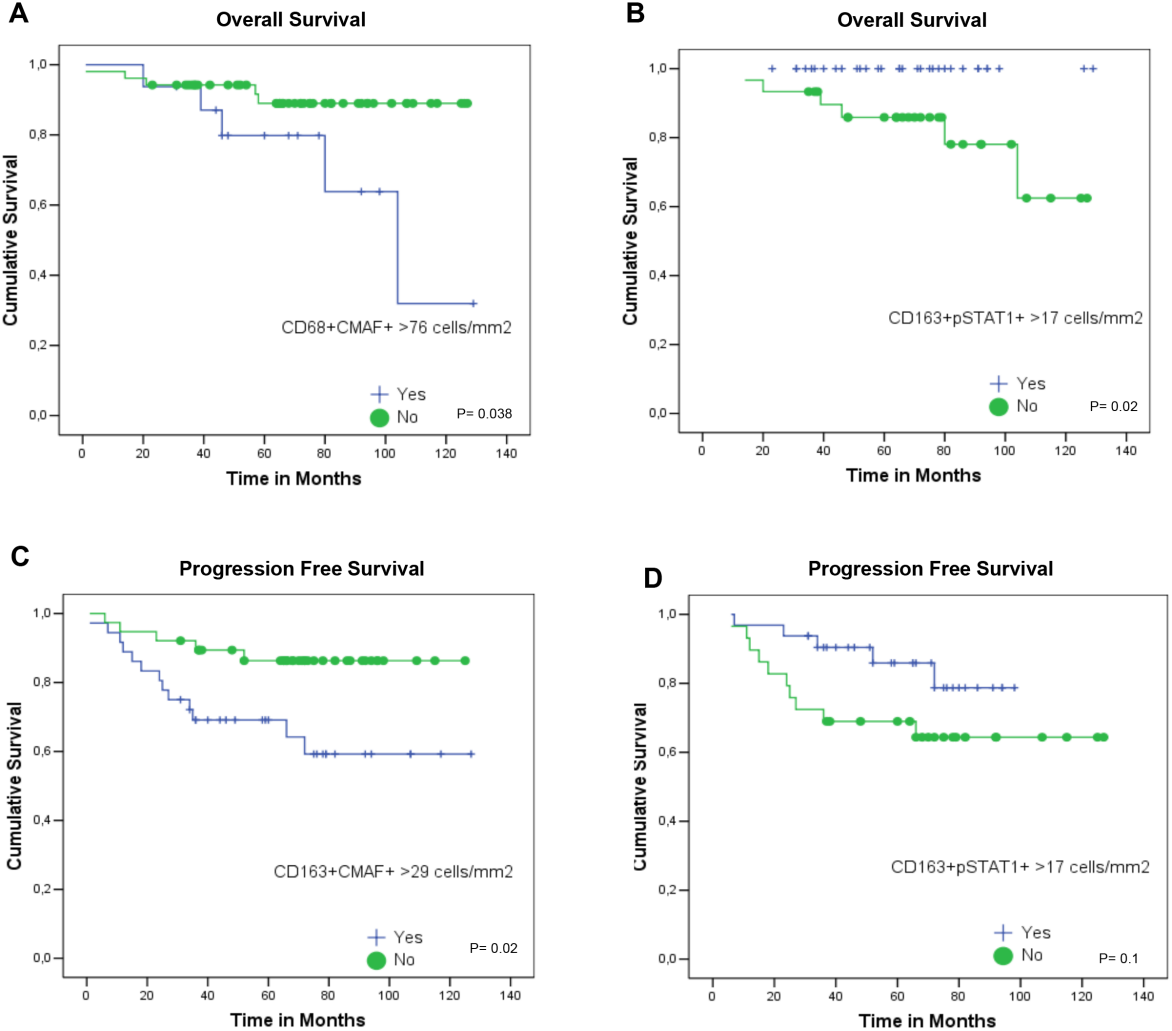
Kaplan-Meier curves in pediatric classical Hodgkin lymphoma, according to number of M1- or M2-like macrophages. A) Overall survival (OS) according to the numbers of CD68+CMAF+ macrophages. B) OS according to the numbers of CD163+pSTAT1+ macrophages. C) Progression-free survival (PFS) according to the numbers of CD163+CMAF+ macrophages. D) PFS according to the numbers of CD163+pSTAT1+ macrophages.

In general, OS and PFS at 120 months were 89.4% and 78.6%, respectively [3]. Considering all cases, worse OS was observed in cases displaying very high numbers of CD68+CMAF+ macrophages (> 76 cells/mm^2^, 75^th^ percentile) (69% vs. 90% for CD68+CMAF+ cells ≤76 cells/mm^2^; *P* = 0.038, Log-rank). On the other hand, better OS was observed in cases with high numbers of CD163+pSTAT1+ macrophages (>17 cells/mm^2^, 50^th^ percentile) (100% vs. 79% for CD163+pSTAT1+ cells ≤17 cells/mm^2^; *P* = 0.02, Log-rank) (Fig 4).

Moreover, M1-like polarization disclosed by CD163+pSTAT1+ / CD163+CMAF+ ratio>1.5 was associated with better OS (100% for M1>M2 vs. 84% for M2>M1. *P* = 0.037, Log-rank).

A worse PFS was observed in cases with high numbers of CD163+CMAF+ macrophages (>29 cells/mm^2^) (64% vs. 87% for ≤29 cells/mm^2^; *P* = 0.02, Log-rank) (Fig 2). High numbers of CD68+pSTAT1+ or CD68+CMAF+ macrophages did not influence the PFS.

## Discussion

Macrophages represent a heterogeneous cell population with functions dependent on their state of polarization [10,11,51]. The absence of universally accepted nomenclature, the dynamics of the activation process and the paucity of suitable macrophage markers are some reasons for the incomplete understanding of the role of macrophages in pathogenesis and outcome of neoplastic diseases [52].

To address this problem, we have recently described an approach using pSTAT1 and CMAF transcription factors together with CD68 and CD163 in double labelling immunohistochemistry to characterise macrophage polarisation in situ [28]. Induction of expression and phosphorylation of STAT1 transcription factor is a well-described early event in macrophage polarization in a Th1-dominant microenvironment [12,19,20]. IFN-gamma signalling induces maximal STAT1 transcription activity as well as phosphorylation of specific STAT1 residues, which facilitates its dimerization, nuclear translocation and DNA binding at IFN-gamma response genes [12,19,20]. Thus, the combined detection of a macrophage marker and pSTAT1 transcription factor is likely to reflect M1 polarization [20].

Several functional studies support the use of CMAF as marker of a subset of M2-polarized macrophages. CMAF is an essential transcription factor that, especially in macrophages, is at the top of the control of IL10 production [22–24]. IL-10 is a Th2 gene product and a potent inhibitor of Th1 cells [53]. IL10 participates in M2 polarization, and M2-polarized macrophages, specifically the M2b subset, produce IL10 [13–17,54,55]. In light of this, CMAF in combination with a macrophage marker (CD68 or CD163) may identify at least a subset of M2-polarized macrophages. Given the importance of IL10 in cHL pathobiology [56–59], we hypothesize that, even if not all M2-polarized macrophages are identified by our approach, CMAF expressing macrophages would represent a biologically significant population in this disease.

It is likely that our approach does not reflect the entire spectrum of macrophage polarization. Nevertheless, previous immunohistochemical studies addressing the role of macrophages in tumour microenvironment have largely relied on the use of CD68 and/or CD163 only and have considered CD163 to be a marker of M2 macrophages. Based on results published here and previously [28] we believe that the latter assumption may be too simple. Our approach is an attempt to characterise macrophage polarization *in situ* in more detail. While it is clear that further studies will be required to validate this approach and that additional marker combinations may be necessary to characterise fully the spectrum of macrophage polarization, we believe that our results add an important piece of information to the understanding of the complexity of lymphoma microenvironment.

In this work, we have used a 1.5 ratio as cut-off to assess predominance of M1 over M2 macrophages and *vice versa*. We acknowledge that this is an arbitrary approach, but this is also the case for other quantitative approaches currently in use such as gene expression analyses [60–62]. Also, using this strategy we were able to identify associations that are in line with our previous results [28] and make sense from an immunological point of view. For instance, we were able to show a comparable macrophage balance in EBV+ cHL and in inflammatory diseases with predominance of cytotoxic/Th1 immune response. Also higher numbers of TIA1+ lymphocytes were observed in cHL cases with more CD68+pSTAT1+ than CD68+CMAF+ macrophages, and higher numbers CD8+ lymphocytes in cases with more CD163+pSTAT1+ than CD163+CMAF+ macrophages.

Differences in the numbers of CD68+ and CD163+ macrophages in the tumor microenvironment of cHL have already been shown by others and by us [4,8,63,64]. It is not clear if the detection of higher numbers of CD68+ cells over CD163+ cells is due to the detection of other cell populations such as dendritic cells by the CD68 PG-M1 clone [65] or if it results from the ability of CD163 antibody to identify macrophage subsets within CD68+ cell population. However, to explore the reasons for these differences was not the objective of this study.

Based on in vitro studies, CD163 has been suggested as a M2 marker [26,27,66]. Furthermore, using CD163 immunohistochemistry, it has been hypothesized that tumor microenvironment in cHL as well as in solid tumors is enriched in M2 macrophages [6–8,67–72]. Here, we show that CD163+ macrophages more often display evidence of M2 polarization than CD68+ macrophages. However, the view of CD163 as a specific M2 marker may represent an oversimplification, not only because a significant proportion of CD163+ macrophages can co-express pSTAT1, which is clearly associated with IFN-g induced M1 polarization [12,29,47,52] but also because CD163+pSTAT1+ macrophages were associated with better survival, suggesting that these cells may have an anti-neoplastic function in cHL.

Furthermore, we show that macrophage polarization mirrors the tumor microenvironment lymphocyte content (cytotoxic vs. immunoregulatory), suggesting that in cHL the relation between a Th1/cytotoxic immune response and M1 polarization, as well as between a Th2/immunoregulatory response and M2 macrophage polarization, described for non neoplastic diseases [73,74], is maintained.

Independently of the nomenclature used, many authors claim that macrophages in the tumor microenvironment of cancer favor immunosuppression and thus contribute to tumor progression [10,11,75,76]. Specifically, it has been suggested that macrophages may be the “bad guys” in cHL [77]. In contrast to this notion, we demonstrate that a cytotoxic/Th1/M1-like profile is prevalent in particular in EBV-associated pcHL. While this is not of the magnitude seen in Th1-predominant inflammatory disorders, it might contribute to the better outcome in this particular clinical subset.

When the survival analyses are considered in the context of the different functional properties of M1 and M2 macrophages [10,11,51,73,74], a differential role of macrophages in pediatric cHL is emerging. In this setting, M1-like macrophages may contribute to the formation of a tumor microenvironment more effective in immune surveillance, while M2-like macrophages may contribute to a dysfunctional microenvironment, not adequate to control the neoplastic proliferation. From this point of view, not all macrophages may be “bad guys” [77]; the use of a single macrophage marker to stratify cHL patients may therefore be inappropriate; and a hypothetical targeted therapy directed against CD68+ or CD163+ cells might have deleterious effects, at least in the group of patients with a M1-predominant microenvironment.

We are aware that the number of cases in this study imposes limitations in relation to the survival analyses, and prospective as well as functional studies are mandatory to confirm these results. Moreover, we acknowledge that the analysis presented here was performed using the same cohort of cases published previously [2–4], and thus requires validation in other case series. However, we believe that our series of sequential cases with similar numbers of EBV+ and EBV- cases is appropriate for the immunological evaluations performed here.

In summary, our results suggest that in pediatric cHL the polarization of macrophages may depend on the tumor microenvironment composition and may be influenced by EBV status of HRS cells. Furthermore, high numbers of M2-like macrophages, but not of M1-like macrophages, are associated with worse OS and PFS.

## Supporting Information

S1 File Supporting Tables. Table A in S1 File: Antibodies used for immunohistochemical study. Table B in S1 File: Clinical, histological and Epstein-Barr virus data at diagnosis. Table C in S1 File: Description of the immune cell populations from the tumor microenvironment previously analyzed. Table D in S1 File: Balance of polarized macrophages according to not-neoplastic diseases with predominance of cytotoxic/Th1 immune response, not-neoplastic diseases with predominance of Th2 immune response and Epstein-Barr virus-associated classical Hodgkin lymphoma. Table E in S1 File: Balance of polarized macrophages according to not-neoplastic diseases with predominance of cytotoxic/Th1 immune response, not-neoplastic diseases with predominance of Th2 immune response and Epstein-Barr virus-negative classical Hodgkin lymphoma. Table F in S1 File: Description of macrophage polarization in the group of not-neoplastic diseases with predominance of cytotoxic/Th1 immune response and in the group of diseases with predominance of Th2 immune response.
(DOC)

S1 Fig Double immunohistochemistry showing the presence of CD4+CMAF+ cells (A), where CD4 is indicated by blue membranous staining and CMAF by nuclear staining. In “B” is shown the presence of CD8+pSTAT1+ cells, where CD8 is indicated by blue membranous staining and pSTAT1 by nuclear staining (original magnification: 400x). The arrows indicate examples of double positive cells. The sections were not counterstained.
(TIF)

S2 Fig Scatter graphs showing the correlation between LYZ expression and STAT1 expression (A); numbers of CD68+pSTAT1+ macrophages and STAT1 expression (B); numbers of CD68+pSTAT1+ macrophages and LYZ expression (C); numbers of CD68+CMAF- macrophages and STAT1 expression (D); numbers of CD68+CMAFmacrophages and LYZ expression; and progression free survival according to the LYZ expression level, using 50th percentile (3.11 fold change) as cut-off (E). (TIF)

S3 Fig Box-plot graphs showing the numerical distribution of CD68+pSTAT1+ macrophages according to age-group (A), nodular sclerosis grade (C) and Epstein-Barr virus association (D), as well as CD163+pSTAT1+ macrophages according to gender (B) and Epstein-Barr virus association (E). The P-value in each bracket is from Mann-Whitney tests.
(TIF)
