# Supplementary material for: Macrophage Polarization Reflects T Cell Composition of Tumor Microenvironment in Pediatric Classical Hodgkin Lymphoma and Has Impact on Survival
Source: PLoS One. 2015 May 15;10(5):e0124531. doi: 10.1371/journal.pone.0124531 (PMC4433187; doi:10.1371/journal.pone.0124531)
Supplement: S1 File — Table A in S1 File: Antibodies used for immunohistochemical study. Table B in S1 File: Clinical, histological and Epstein-Barr virus data at diagnosis. Table C in S1 File: Description of the immune cell populations from the tumor microenvironment previously analyzed. Table D in S1 File: Balance of polarized macrophages according to not-neoplastic diseases with predominance of cytotoxic/Th1 immune response, not-neoplastic diseases with predominance of Th2 immune response and Epstein-Barr virus-associated classical Hodgkin lymphoma. Table E in S1 File: Balance of polarized macrophages according to not-neoplastic diseases with predominance of cytotoxic/Th1 immune response, not-neoplastic diseases with predominance of Th2 immune response and Epstein-Barr virus-negative classical Hodgkin lymphoma. Table F in S1 File: Description of macrophage polarization in the group of not-neoplastic diseases with predominance of cytotoxic/Th1 immune response and in the group of diseases with predominance of Th2 immune response. (DOC) [file pone.0124531.s001.doc]

**Supporting Information 1**

Table A: Antibodies used for immunohistochemical study.

| **Antibody** | **Clone** | **Source** | **Dilution** | **Buffer Retrieval** |
| --- | --- | --- | --- | --- |
| pSTAT1 | Polyclonal | Santa Cruz | 1:100 | EDTA |
| CMAF | M-153 | Santa Cruz | 1:50 | EDTA |
| CD68 | PGM1 | Dako | 1:1500 | EDTA |
| CD163 | 10D6 | Novocastra | 1:3000 | EDTA |

Table B: Clinical, histological and Epstein-Barr virus data at diagnosis.

| **Variable** | **Cases Analyzed (%)** |
| --- | --- |
| Age, years  < 14  > 14 | 57/100 (57)  43/100 (43) |
| Sex  Male  Female | 64/100 (64)  36/100 (36) |
| Stage  I  II  III  IV^1^ | 11/95 (11.6)  48/95 (50.5)  25/95 (26.3)  11/95 (11.6) |
| Extranodal disease  Yes  No | 11/95 (11.6)  84/95 (88.4) |
| B symptoms  Yes  No | 51/95 (53.7)  44/95 (46.3) |
| Clinical Presentation  Favorable  Unfavorable | 50/95 (52.6)  45/95 (47.4) |
| Leukopenia  Yes  No | 11/96 (11.5)  85/96 (88.5) |
| Histopathological Diagnosis  Nodular sclerosis  Mixed cellularity  Lymphocyte-depleted  Lymphocyte-rich  Unclassifible | 69/100 (69)  23/100 (23)  1/100 (1)  2/100 (2)  5/100 (5) |
| EBV Association  Yes  No | 43/96 (44.8)  53/96 (55.2) |

^1^Lung compromised in 5 children; pericardium compromised in 2 children; long bones compromised in 2 children; bone marrow compromised in 2 children; adrenal compromised in 1 child and liver compromised in 1 child. EBV: Epstein-Barr virus.

Table C: Description of the immune cell populations from the tumor microenvironment previously analyzed.

| **Variable** | **Labeled Cells /mm^2^** | **Cases Analyzed (%)** |
| --- | --- | --- |
| **CD3 (cells/mm^2^)**  Range  (Mean / Median) | 70 to 1187  (643.43 / 645) |  |
| < 451 (25^th^ percentile) |  | 22/83 (26.5) |
| > 451 (25^th^ percentile) |  | 61/83 (73.5) |
| < 645 (50^th^ percentile) |  | 42/83 (50.6) |
| > 645 (50^th^ percentile) |  | 41/83 (49.4) |
| **CD4 (cells/mm^2^)**  Range  (Mean / Median) | 1 to 624  (180.02 / 155) |  |
| < 70 (25^th^ percentile) |  | 21/83 (25.3) |
| > 70 (25^th^ percentile) |  | 62/83 (74.7) |
| < 155 (50^th^ percentile) |  | 42/83 (50.6) |
| > 155 (50^th^ percentile) |  | 41/83 (49.4) |
| **FOXP3 (cells/mm^2^)**  Range  (Mean / Median) | 1 to 513  (101.64 / 49) |  |
| < 12 (25^th^ percentile) |  | 21/84 (25) |
| > 12 (25^th^ percentile) |  | 63/84 (75) |
| < 49 (50^th^ percentile) |  | 44/84 (52.4) |
| > 49 (50^th^ percentile) |  | 40/84 (47.6) |
| **TBET (cells/mm^2^)**  Range  (Mean / Median) | 2 to 269  (54.93/ 32) |  |
| < 15.5 (25^th^ percentile) |  | 20/81 (24.7) |
| > 15.5 (25^th^ percentile) |  | 61/81 (75.3) |
| < 32 (50^th^ percentile) |  | 41/81 (50.6) |
| > 32 (50^th^ percentile) |  | 40/81 (49.4) |
| **CD8 (cells/mm^2^)**  Range  (Mean / Median) | 11 to 847  (185.26 / 143) |  |
| < 76 (25^th^ percentile) |  | 22/86 (25.6) |
| > 76 (25^th^ percentile) |  | 64/86 (74.4) |
| < 143 (50^th^ percentile) |  | 44/86 (51.2) |
| > 143 (50^th^ percentile) |  | 42/86 (48.8) |
| **TIA1 (cells/mm^2^)**  Range  (Mean / Median) | 1 to 396  (113.64 / 69) |  |
| < 39 (25^th^ percentile) |  | 20/81 (24.7) |
| > 39 (25^th^ percentile) |  | 61/81 (75.3) |
| < 69 (50^th^ percentile) |  | 41/81 (50.6) |
| > 69 (50^th^ percentile) |  | 40/81 (49.4) |
| **Granzyme B (cells/mm^2^)**  Range  (Mean / Median) | 1 to 451  (32.07 / 11) |  |
| < 4 (25^th^ percentile) |  | 24/85 (28.2) |
| > 4 (25^th^ percentile) |  | 61/85 (71.8) |
| < 11 (50^th^ percentile) |  | 44/85 (51.8) |
| > 11 (50^th^ percentile) |  | 41/85 (48.2) |
| **CD20 (cells/mm^2^)**  Range  (Mean / Median) | 1 to 885  (240.86 / 196) |  |
| < 79 (25^th^ percentile) |  | 21/83 (25.3) |
| > 79 (25^th^ percentile) |  | 62/83 (74.7) |
| < 196 (50^th^ percentile) |  | 42/83 (50.6) |
| > 196 (50^th^ percentile) |  | 41/83 (49.4) |
|  |  |  |
|  |  |  |

Table D: Balance of polarized macrophages according to not-neoplastic diseases with predominance of cytotoxic/Th1 immune response, not-neoplastic diseases with predominance of Th2 immune response and Epstein-Barr virus-associated classical Hodgkin lymphoma.

|  | **MACROPHAGE BALANCE** | **Diseases with predominance of cytotoxic/Th1 immune response**  **(%)** | **Diseases with predominance of Th2 immune response**  **(%)** | **EBV+ cHL**  **(%)** | **P** |
| --- | --- | --- | --- | --- | --- |
|  | **CD68+pSTAT1+ : CD68+CMAF+ cells^a^** |  |  |  |  |
|  | M1 > M2 | 25 (89.3) | 0 | 20 (64.5) |  |
|  | M2 > M1 | 1 (3.6) | 40 (100) | 11 (35.5) |  |
|  | M1 ≈ M2 | 2 (7.1) | 0 | 0 |  |
|  | Total | 28 (100) | 40 (100) | 31 (100) | < 0.0005 |
|  | **CD163+pSTAT1+ : CD163+CMAF+ cells^b^** |  |  |  |  |
|  | M1 > M2 | 18 (64.3) | 0 | 14 (50) |  |
|  | M2 > M1 | 3 (10.7) | 40 (100) | 11 (39.3) |  |
|  | M1 ≈ M2 | 7 (25) | 0 | 3 (10.7) |  |
|  | Total | 28 (100) | 40 (100) | 28 (100) | < 0.0005 |

a) Considering CD68 as macrophage marker; ratio between the numbers of CD68+pSTAT1+ macrophages (M1) and CD68+CMAF+ macrophages (M2); b) Considering CD163 as macrophage marker; ratio between the numbers of CD163+pSTAT1+ macrophages (M1) and CD163+CMAF+ macrophages (M2). EBV+ cHL: Epstein-Barr virus-associated classical Hodgkin lymphoma.

Table E: Balance of polarized macrophages according to not-neoplastic diseases with predominance of cytotoxic/Th1 immune response, not-neoplastic diseases with predominance of Th2 immune response and Epstein-Barr virus-negative classical Hodgkin lymphoma.

|  | **MACROPHAGE BALANCE** | **Diseases with predominance of cytotoxic/Th1 immune response**  **(%)** | **Diseases with predominance of Th2 immune response**  **(%)** | **EBV- cHL**  **(%)** | **P** |
| --- | --- | --- | --- | --- | --- |
| **Comparison with EBV- cHL cases** | **CD68+pSTAT1+ : CD68+CMAF+ cells^a^** |  |  |  |  |
|  | M1 > M2 | 25 (89.3) | 0 | 21 (53.8) |  |
|  | M2 > M1 | 1 (3.6) | 40 (100) | 16 (41) |  |
|  | M1 ≈ M2 | 2 (7.1) | 0 | 2 (5.1) |  |
|  | Total | 28 (100) | 40 (100) | 39 (100) | < 0.0005 |
|  | **CD163+pSTAT1+ : CD163+CMAF+ cells^b^** |  |  |  |  |
|  | M1 > M2 | 18 (64.3) | 0 | 13 (39.4) |  |
|  | M2 > M1 | 3 (10.7) | 40 (100) | 20 (60.6) |  |
|  | M1 ≈ M2 | 7 (25) | 0 | 0 |  |
|  | Total | 28 (100) | 40 (100) | 33 (100) | < 0.0005 |

a) Considering CD68 as macrophage marker; ratio between the numbers of CD68+pSTAT1+ macrophages (M1) and CD68+CMAF+ macrophages (M2); b) Considering CD163 as macrophage marker; ratio between the numbers of CD163+pSTAT1+ macrophages (M1) and CD163+CMAF+ macrophages (M2). EBV- cHL: Epstein-Barr virus-negative classical Hodgkin lymphoma.

Table F: Description of macrophage polarization in the group of not-neoplastic diseases with predominance of cytotoxic/Th1 immune response and in the group of diseases with predominance of Th2 immune response.

| **MACROPHAGE BALANCE** | **Diseases with predominance of cytotoxic/Th1 immune response**  **(%)** | **Diseases with predominance of Th2 immune response**  **(%)** | **P** |
| --- | --- | --- | --- |
| **CD68+pSTAT1+ : CD68+CMAF+ cells^a^** |  |  |  |
| M1 > M2 | 25 (89.3) | 0 |  |
| M2 > M1 | 1 (3.6) | 40 (100) |  |
| M1 ≈ M2 | 2 (7.1) | 0 |  |
| Total | 28 (100) | 40 (100) | < 0.0005 |
| **CD163+pSTAT1+ : CD163+CMAF+ cells^b^** |  |  |  |
| M1 > M2 | 18 (64.3) | 0 |  |
| M2 > M1 | 3 (10.7) | 40 (100) |  |
| M1 ≈ M2 | 7 (25) | 0 |  |
| Total | 28 (100) | 40 (100) | < 0.0005 |

a) Considering CD68 as macrophage marker; ratio between the numbers of CD68+pSTAT1+ macrophages (M1) and CD68+CMAF+ macrophages (M2); b) Considering CD163 as macrophage marker; ratio between the numbers of CD163+pSTAT1+ macrophages (M1) and CD163+CMAF+ macrophages (M2).
