# Supplementary figures and images for: Macrophage Polarization Reflects T Cell Composition of Tumor Microenvironment in Pediatric Classical Hodgkin Lymphoma and Has Impact on Survival
Source: PLoS One. 2015 May 15;10(5):e0124531. doi: 10.1371/journal.pone.0124531 (PMC4433187; doi:10.1371/journal.pone.0124531)

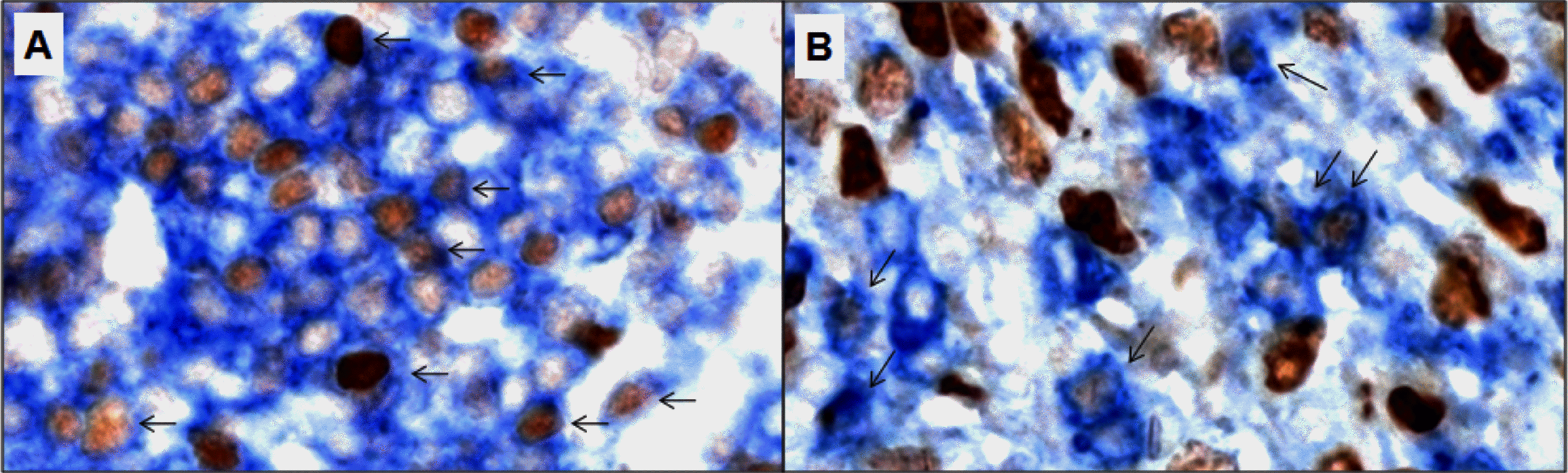

Supplement: S1 Fig — In “B” is shown the presence of CD8+pSTAT1+ cells, where CD8 is indicated by blue membranous staining and pSTAT1 by nuclear staining (original magnification: 400x). The arrows indicate examples of double positive cells. The sections were not counterstained. (TIF) [file pone.0124531.s002.tif]

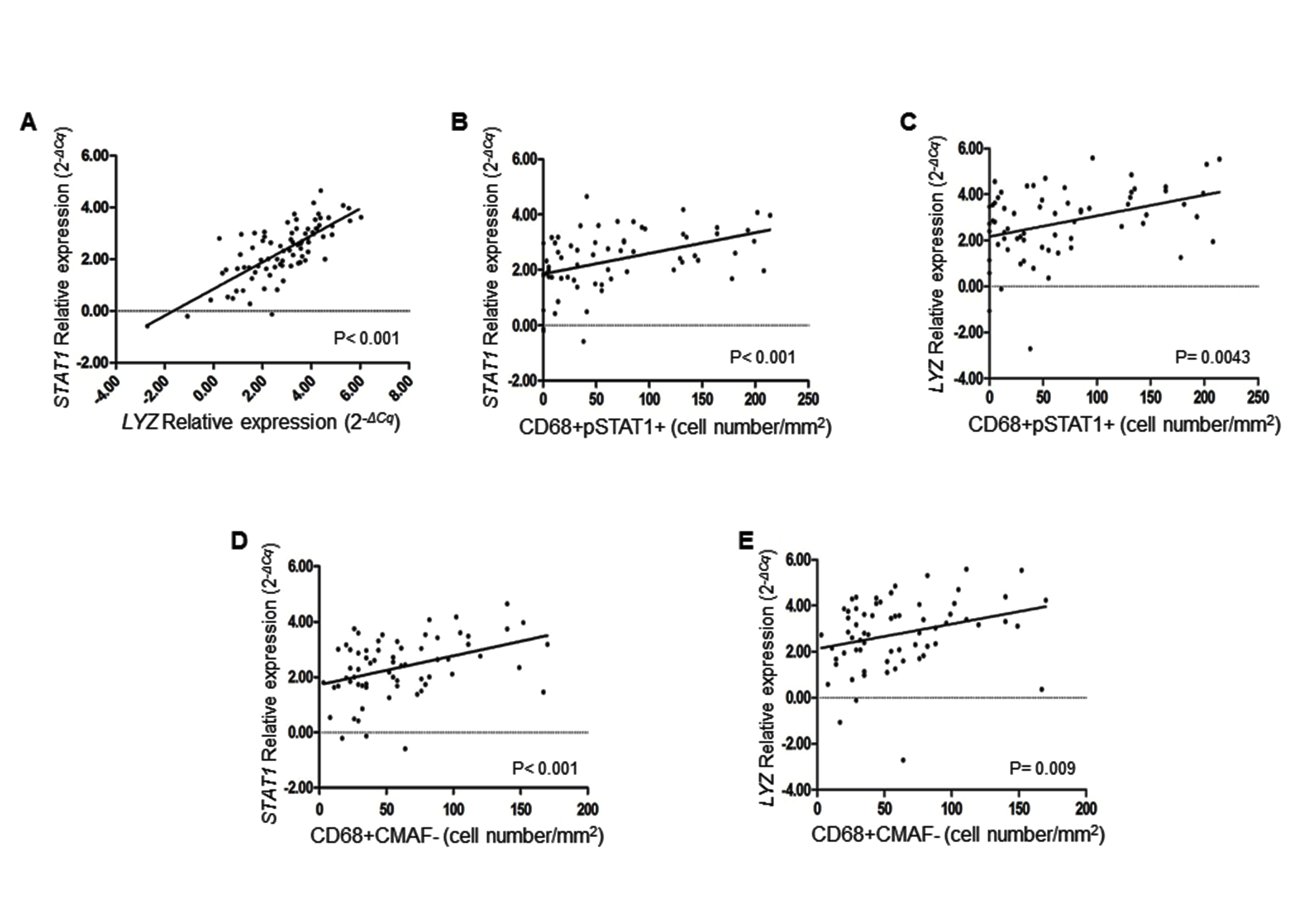

Supplement: S2 Fig — (TIF) [file pone.0124531.s003.tif]

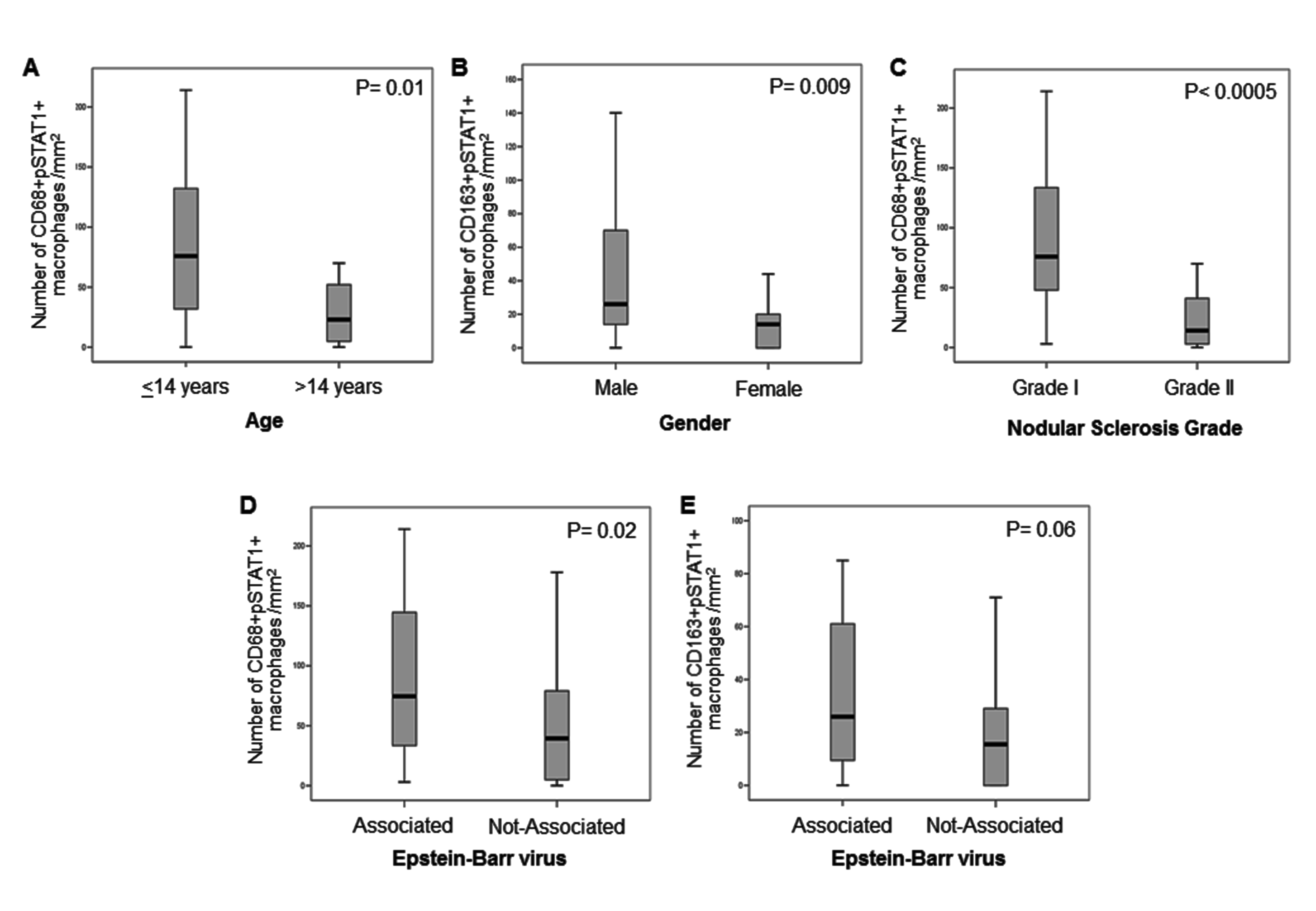

Supplement: S3 Fig — The P-value in each bracket is from Mann-Whitney tests. (TIF) [file pone.0124531.s004.tif]
